# Nutritional Quality of Rye Bread with the Addition of Selected Malts from Beans

**DOI:** 10.3390/molecules30051006

**Published:** 2025-02-21

**Authors:** Anna Czubaszek, Mateusz Gertchen, Alan Gasiński, Joanna Miedzianka, Joanna Kawa-Rygielska

**Affiliations:** 1 Department of Fermentation and Cereals Technology, Wrocław University of Environmental and Life Sciences, 50-375 Wrocław, Poland; anna.czubaszek@upwr.edu.pl (A.C.); alan.gasinski@upwr.edu.pl (A.G.); joanna.kawa-rygielska@upwr.edu.pl (J.K.-R.); 2 Department of Food Storage and Technology, Wrocław University of Environmental and Life Sciences, 50-375 Wrocław, Poland; joanna.miedzianka@upwr.edu.pl

**Keywords:** rye bread, legumes, white bean, red bean, malts, antioxidant activity

## Abstract

This study aimed to evaluate the effect of partial rye flour (RF) replacement with white bean malt (WBM) and red bean malt (RBM) on the baking and the nutritional quality of bread. The addition of white and red bean malts to the rye flour reduced the falling number and the maximum viscosity of the paste. Significant differences in the color of the crust and crumb of baked bread were shown. The addition of malt from bean seeds did not cause significant changes in the consumer assessment of bread. In some cases, a 30% increase in the polyphenols content was observed and an improvement in the antioxidant properties of bread with WBM and RBM was noted. Also, the overall protein and essential amino acids content in the bread was significantly increased. Due to WBM and RBM addition, the quantity of volatile compounds was higher than it was in the control sample, and in specific instances, it had doubled compared to the control sample.

## 1. Introduction

Cereal products play an important role in the human diet. They are considered the most common among products of plant origin due to their relatively low price and easy availability. Cereal grains are the most important source of energy supplied to the body with food [1]. They play a significant role in providing vegetable protein. In addition, they are also a good source of vitamins, especially from group B and vitamin E as well as minerals such as calcium, magnesium, potassium, phosphorus, iron, and sodium [2,3]. Among the cereal products, bread is one of the most consumed articles worldwide, and in northern Europe, rye bread is particularly popular [4,5,6].

The growing nutritional awareness of consumers makes them look for food products that, apart from sensory qualities, have a beneficial effect on health [2]. Consumers give up products that are not sufficiently valuable from a nutritional point of view. Therefore, to meet the consumer’s expectations and needs, producers are trying to expand their offer with products characterized by a higher nutritional value and appropriate palatability. For this purpose, in bread production, various raw materials and natural additives are used more and more often, and are the source of specific nutrients and have a health-promoting effect [7,8,9].

One of these kinds of raw materials is legume seeds. They stand out in comparison to other plants, mainly due to their high protein content. Combining legume seeds with cereal grains makes it possible to obtain a product with wholesome protein [10,11]. In addition, legume seeds are a source of dietary fiber, minerals, vitamins, and compounds with a potential antioxidant effect [12,13,14,15,16]. The most important legume plant used for consumption in the world is the common bean (*Phaseolus vulgaris* L.) with its numerous varieties, among them, white and red bean [17]. Colored beans contain more flavonoids than white beans [18,19]; however, both of them are also the source of anti-nutritional substances such as α-galactosides phytic acid, tannins, and trypsin inhibitors [20,21]. Due to this, preparing beans and seeds or other legumes for consumption requires the use of specific processing methods that will reduce the content of anti-nutritional ingredients and improve the bioavailability and digestibility of protein, vitamins, and minerals. One of the processes used to modify the physical structure of grains and activate enzymes in them is malting. During this technological process, the goal of which is to produce more fermentative substance, a grain is alternatively submerged in water and stored in humid air to increase the moisture, then, grains are germinated and dried in ovens [22]. Although it is mainly used to modify grains of barley (*Hordeum vulgare*), the malting of legumes has been successfully carried out [23]. Germination, which is part of the malting process, has a positive effect on legume seeds. It has been shown that the use of this method increases the concentration of polyphenols and improves antioxidant capacity and the bioavailability of protein, vitamins, and minerals in seeds [24,25,26]. There are reports concerning sprouted beans produced in the germination process, which were added to bread—mainly wheat bread [27,28,29]. These studies proved that sprouted beans can impact the protein content in bread and dough rheological properties. The use of this additive can also increase the volume of dough during fermentation. The advantage of the malting process over germination is positive changes in the organoleptic characteristics and a greater stability of malt compared to germinated seeds [30].

The available literature lacks information on the influence of the addition of bean malt on the properties of rye bread; therefore, research was undertaken to compare the influence of the addition of white and red bean malt to rye flour on selected baking characteristics of mixtures of rye flour with bean malt and the nutritional quality of baked bread.

## 2. Results and Discussion

The falling number and amylographic maximum viscosity are used to determine the activity of amylolytic enzymes and the susceptibility of starch to their action in the flour and, thus, determine the suitability of the flour for baking bread. Figure S1a,b (Supplementary Materials) illustrates the results of the falling number and maximum viscosity. It was found that rye flour used in the research was characterized by a low activity of amylolytic enzymes and good properties of the amylase-starch complex (falling number—234 s; maximum viscosity—690 AU). Michalska et al. [31] state that rye flour intended for the production of bread should have a falling number ranging from 125 to 200 s, and according to the PN-A-74032:2002 [32] standard, the falling number of rye flour should range from 90 to 240 s. In our research, the falling number of rye flour and its mixtures with WBM and RBM was within the reported standard range or slightly higher (Figure S1a—Supplementary Materials). It was found that the increasing share of WBM in the mixture with rye flour resulted in a successive decrease in the falling number. The introduction of RBM into rye flour did not cause changes in the discussed parameter or result in its increase. Stępniewska et al. [33] reported that the maximum viscosity of various rye flours commercially available on the Polish market ranges from 410 AU to 940 AU and depends on the flour extract and environmental conditions prevailing during the growth of the rye. According to the mentioned authors, the average viscosity of low-extraction flours is 510 AU. Rye flour used in our own research and samples with various amounts of WBM and RBM were characterized by a maximum viscosity in the range of 375–690 AU (Figure S1b—Supplementary Materials). Based on the amylographic analysis, a successive deterioration of the rheological properties of the paste was observed due to the increase in the share of WBM and RBM in the mixture with rye flour. RBM had a smaller impact on the maximum viscosity of the paste than WBM. However, it should be noted that all tested samples were characterized by good technological value assessed based on the falling number and maximum viscosity.

**Figure 1:**
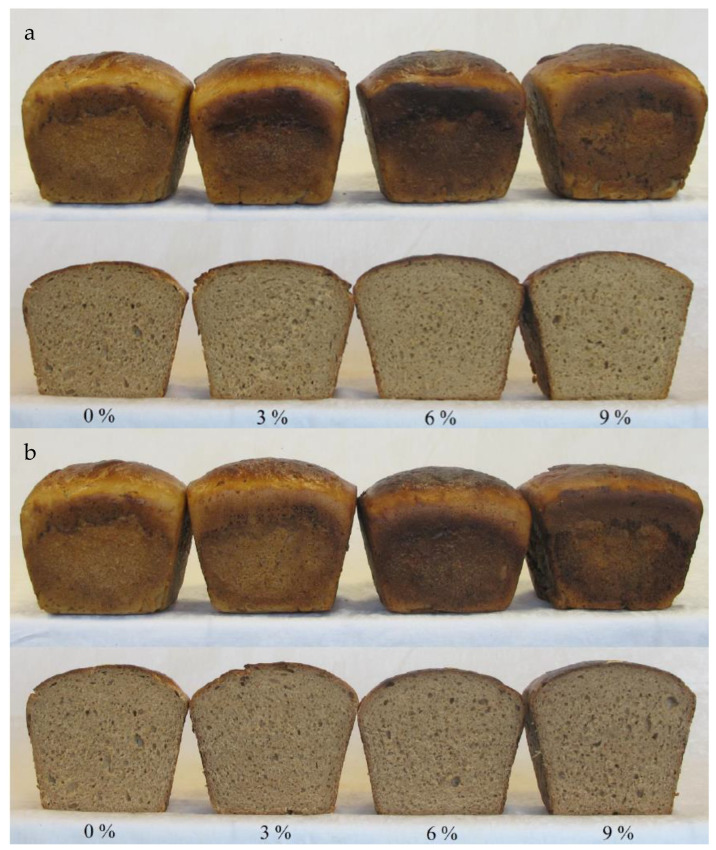
The appearance of rye bread without any addition and with the addition of (**a**) white bean malt (WBM) and (**b**) red bean malt (RBM).

The appearance of the product has a key impact on the decisions made by consumers. Obtained breads were characterized by the right shape and volume. Baked breads with the addition of WBM and RMB (Figure 1) were properly risen. The obtained bread had a fairly uniform crumb structure. The crust of the bread did not stick out from the crumb and was dark brown. The crumb of the bread was elastic and quite evenly porous. It was noted that the porosity of breads with WBM was rated slightly higher than that of breads with RBM. Rizzello et al. [34] observed that the addition of legume flour to wheat bread production in amounts of 22.5% and 30% caused a negative effect on the structure. Our research also shows a slight tendency for porosity to deteriorate with a 9% addition of bean malts.

Affected by malt addition, the bread yield varied from 160.2% (6% RBM) to 163.4% (3% WBM) (Table 1).

Observed differences were not statistically significant; however, a 1–3% higher bread yield with 3–9% WBM addition and 3% RMB addition can have significant economic importance for production on an industrial scale. When assessing the volume of the bread from 100 g of flour it was found that the biggest and most statistically significantly difference was that between the rye bread with 9% RBM (431.7 cm^3^) and rye bread without any addition (401.7 cm^3^). The remaining breads had a volume in the range of 414.2–422.5 cm^3^. A tendency for the volume to increase with the increase in the share of malt in the bread was observed. It may be the result of the malting process. During this process, the activity of amylases increases [35], and after adding malted materials to the dough, the availability of sugars may increase, which has a positive effect on the fermentation process and may cause the dough to expand more. Aguilar et al. [36] observed that the malting process of quinoa caused an increased level of reducing sugars. Observed changes in the volume of breads containing bean malt may be the result of the elevated content of simple sugars in the malted raw material, which has a beneficial effect on the fermentation process and may cause greater dough expansion. Ukeyima et al. [37] obtained different results. The mentioned authors have demonstrated the negative influence of sprouted, cooked, and then dried white been seeds addition on wheat bread volume. However, the bean seeds used in that study were processed differently than the seeds in our study, and their share in the bread was higher and amounted to 10, 15, 20, and 25%.

Color is an important determinant of the attractiveness of food products. Table 1 shows the bread crumb and crust color parameters. Assessing the color of the bread crust, it was shown that breads containing RBM had a crust characterized by L* values similar to those of the control sample, and at all levels of WBM addition, there was a reduction in the value of the L* parameter, which determines the lightness of the colors. Therefore, the crust of breads containing WBM has a darker color compared to the other samples. The results of the a* parameter represent the area between the intensity of red and green. The highest share of red color was found in the crust of rye bread without any addition (a* = 9.73) and rye bread with 3% RBM (a* = 9.37), and the lowest was found in the crust of bread with 6% and 9% addition of WBM (a* = 6.14 and 6.60, respectively). The b* parameter determines the color changes from blue to yellow. The highest share of yellow color was observed in the crust of the control sample (b* = 9.14) and rye bread with 3% RBM addition (b* = 8.47). A smaller share of this color in the crust was observed in breads containing WBM compared to those containing RBM.

By assessing the color of the bread crumb, it was determined that the addition of WBM did not have a statistically significant effect on the average value of the L* parameter. Taking into account the share of RBM, it was shown that the higher it was, the darker the crumb color of the baked loaf. Based on the average values of parameters a* and b*, it was found that with the increase in the addition of RBM, the color of the bread crumb was characterized by a smaller share of yellow and a bigger share of red. The addition of WBM increased the share of yellow color in the crumb, and the highest share of this color was recorded in the case of 9% of the addition (b* = 19.63). The value of the a* parameter of breads with this addition, compared to rye bread, decreased significantly only with 9% malt content. All a* and b* values obtained when assessing the color of the crust and crumb of baked bread were positive numbers, which means the lack of green and blue color in the analyzed samples, respectively.

Manonmani et al. [38] determined the color on the L*a*b* scale in wheat bread with various additions of dried and ground red bean seeds. They observed a decrease in the value of the L* parameter and b* parameter and an increase in the value of the a* parameter. In the cited studies, the bread crumb, with an increase in the share of red bean seeds, was characterized by an increasingly darker color and a greater share of red and a smaller share of yellow. In our research, the addition of RBM had the same effect on the color of the crumb. A different effect on the color of the bread crumb was observed when WBM was used, because its content did not affect the changes in the value of the L* parameter or the a* parameter at 3% and 6% addition; however, it increased the value of the b* parameter.

The water content in bread is important information for both nutritional and organoleptic reasons because consumers often equate the moisture of the crumb with the freshness of the bread. The obtained results indicate that the moisture ranged from 47.35 (6% RBM) to 48.75 g/100 g (9% RBM) (Table 2). It was found that the bean malt additives used did not statistically significantly change the value of this feature compared to the control sample (48.15 g/100 g).

Minerals play an important role in building the body and participate in the proper course of many reactions and processes. Their content in bread depends on the flour used for baking; the lighter the flour, the lower the amount of minerals it contains. Ash is the sum of minerals contained in the product. The average content of these ingredients in the tested breads did not vary much and ranged from 0.69 to 0.78 g/100 g (Table 2). A significant difference was found only between bread with 3% and 9% RBM. The least minerals were contained in bread with 3% RBM (0.69 g/100 g), and the most in bread with 9% RBM (0.78 g/100 g). The 3–9% malt additions used in the studies were probably too small to significantly affect changes in the amount of minerals. Mariscal-Moreno et al. [17] observed that a 10% addition of black beans to bread did not increase the ash content in bread. Only the additions of 20% and 30% significantly increased the ash content in the tested bread.

The fat in bread mainly comes from the cereals from which the flour used for baking are produced. The fat content in the baked breads ranged from 0.87 to 1.02 g/100 g (Table 2). The highest amount of this nutrient was contained in bread with 9% WBM and the lowest in bread with 6% RBM. The differences between the remaining breads in terms of fat content were statistically insignificant. According to USDA “https://fdc.nal.usda.gov/fdc-app.html#/food-search” (accessed on 20 December 2024), rye flour, white beans, and red beans have a similar fat content (respectively, 1.91, 1.32, and 1.16 g/100 g) and are not a rich source of this ingredient. Therefore, the partial replacement of rye flour by WBM or RBM does not significantly affect the fat content in bread.

Beans are a valuable source of plant protein. It was found in our study that as the addition of WBM and RBM to rye flour increased, the total protein content in baked bread increased (Table 2). Rye bread without added malt contained the lowest amount of protein, at 4.13 g/100 g. Compared to the control sample, the highest increase in protein content, amounting to approximately 20%, was recorded in bread with 9% WBM (4.98 g/100 g). The same addition of RBM increased the amount of this ingredient by 15% compared to the control. Ukeyima et al. [37] also observed that as the proportion of ground white bean seeds increased, the total protein content in baked bread increased. Bhol and John Don Bosco [39] added red kidney bean flour to blend with wheat flour. They observed that the substitution of 20 g of red kidney bean flour for wheat flour doubled the protein content in the tested bread.

Starch is a carbohydrate that is one of the most important components of rye flour. As the share of malts in rye flour increased, its content in bread decreased slightly (Table 2). There was 36.29 g of starch in 100 g of rye bread. In breads with WBM, a significant reduction in the content of the ingredient in question was found with just 3% of the content, while in breads with RBM, a significant difference was found only with its 9% share in the bread. Based on USDA data posted on the FoodData Central Search Results website “https://fdc.nal.usda.gov/fdc-app.html#/food-search” (accessed on 20 December 2024), the content of starch in rye flour is higher than in white and red beans, so the reduction in starch content in bread with WBM and RBM is understandable. There are no data in the literature regarding the influence of the malting process on the starch content in beans. However, Farzaneh et al. [40] observed that during germination, which is one of the malting stages, the starch content in barley grains decreased significantly. Presumably, this tendency also occurs during the malting of beans, so the reduction in starch content in bread is fully explainable.

The content of total dietary fiber was in the range of 10.26–11.36 g/100 g (Table 2). It was shown that the addition of both WBM and RBM increased the amount of total dietary fiber in bread compared to the control sample. In the study conducted by Ukeyima et al. [37], it was found that the addition of white bean seeds increased the crude fiber content compared to the control sample. These results are also consistent with those obtained by Stoin et al. [41]. According to them, replacing rice flour with 10, 20, 30, and 40% additions of red bean flour was correlated with an increase in the fiber content of breads baked with these additions.

Based on the amino acid profile of rye bread enriched with white and red bean malt, it can be concluded that the bread with 9% addition of both ingredients attained the highest sum of all amino acids (82.95–84.77 mg/g) compared to the control sample (66.09 mg/g) (Table 3).

Breads with WBM contained a higher level of essential amino acids, ranging from 24.42 to 33.66 mg/g, regardless of the amount of malt introduced, than the control sample (22.04 mg/g). However, bread with RBM showed a higher level of the sum of essential amino acids only when higher amounts were used, i.e., 6% and 9% of malt (26.85–28.24 mg/g). The dominant essential amino acids in the analyzed enriched rye breads were leucine, lysine, and phenylalanine. Sulfur amino acids (methionine and cysteine) were present in the lowest amounts, regardless of the sample. In all analyzed samples, the dominant amino acid was glutamic acid, and its content ranged from 20.99 mg/g (control sample) to 26.26 mg/g (breads with 9% RBM). The use of white bean malt when baking rye bread at a dose of just 3% contributed to increasing the content of all amino acids and, thus, increasing the nutritional value of the finished product, compared to the control sample. Gambuś et al. [42] also observed that the dominant amino acid in rye flour (type 720) is glutamic acid.

Consumers are increasingly paying attention to the health-promoting properties of products. Polyphenols are compounds with health-beneficial properties. The addition of WBM and RBM resulted in an equal increase in the total polyphenol content in baked breads (Table 4).

The higher the share of each malt, the greater the amount of these compounds that was determined. Antioxidant activity in breads was determined against the ABTS cation radical, the DPPH radical, and the FRAP method. It was shown that bread with RBM had a higher activity compared to the activity of control bread and bread with WBM and the malt content in the bread was at the same level (Table 4). The addition of WBM improved the antioxidant properties compared to the control bread, but to a lesser extent than was shown with the addition of RBM. Due to the content of polyphenols in beans and the positive impact of the malting process on their concentration, it is not surprising that the polyphenol content and antioxidant activity in bread with the addition of malted beans is higher compared to the control bread. One of the factors that could limit this effect is the high temperature during baking [43]. There are no data in the literature regarding the application of malted beans to rye bread. However, Gallegos-Infante et al. [44] reported that the addition of Mexican common bean flour to the pasta dough increased the content of polyphenols in the final product. Also, Anton et al. [45] observed that the addition of bean flour to tortilla dough caused a higher content of polyphenols and increased antioxidant activity compared to the tortillas prepared from the 100% wheat dough. In both the pasta and tortilla production processes, a high temperature is used.

Gas chromatography and mass spectroscopy allowed identifying and quantifying a total of 34 different volatile compounds in breads with the addition of bean malts (Table 5).

It can be seen that breads baked with the addition of the bean malts were characterized by an increased concentration of the most of detected volatiles. Malting increases the activity of various classes of enzymes in grains and seeds, such as amylases, proteases, lipases, xylanases, cellulases, and lipooxygenases (LOX) [35]. Aldehydes are one of the main volatiles present in most of the bread types and aldehydes are typically formed from fatty acids due to the complex enzymatic reactions started by the activity of LOX [46]. It has been previously proven that malting of the grains can increase the amount of aldehydes in the malt, as well as influence the concentration of aldehydes in the product created from malt [47]. It was detected in the previous studies on malted legumes that other seeds from the Leguminosae family (lentils) were also characterized by a far greater concentration of aldehydes than unmalted seeds [48]. The increased concentration of the aldehydes in the breads with addition of the bean malts is, therefore, most probably caused by two different factors: increased activity of the LOX enzymes, and the fact that the added malt was most probably characterized by a high aldehyde content. Another group of compounds that is important in formation of the bread aroma is furans, which are formed in the process of bread baking through the so-called Maillard reaction [49]. The only compound from this group, a 2-pentyl furan, is one of the most commonly identified furans in various kinds of bread products [50]. Various other components formed during baking in the Maillard reaction were present in the breads produced with the addition of the bean malts analyzed in this study in a higher quantity than in the control samples. Two of the main factors mentioned before, interconnected with each other, are the most possible culprits for this phenomenon. The addition of malts increases the quantity of various enzymatic reactions in the bread dough. As a result, the bread dough should contain a higher concentration of various products of enzymatic reactions, such as the aforementioned aldehydes, but more importantly, a reduction in sugars, free fatty acids, and amino acids. At the first stages of the bread baking, reactions similar to those observed during wort brewing (in which, typically, malts are used) occur: Enzymatic reactions are accelerated and a higher concentration of various products is created. At the same time, these components undergo Maillard reactions, significantly altering the composition of the volatiles present in the bread [35,49,50]. Unfortunately, without precise measurements of the concentrations of particular volatiles throughout the bread-making process, it is impossible to reliably confirm these results and to determine which particular factors influence the concentrations of various volatiles in the finished bread. However, it opens an interesting avenue of research for the future, which can be further expanded by the variety of malts that can be used in the processes of the bread production. Notwithstanding, the results of this study show clearly that addition of various bean malts to the production of the rye breads can be successfully utilized in increasing the concentration of volatiles typical for the aroma of bread, potentially improving the flavor of the finished product.

The bread baked during the research should be attractive to consumers in terms of specific sensory characteristics. For this purpose, the breads are subjected to consumer evaluation. In the study, a 9-point hedonic scale was used to conduct an assessment. All additions of WBM and RBMs to rye bread did not affect the results of consumer assessment in terms of crumb color, crumb consistency and texture, taste, smell, and overall desirability (Table 6).

The mentioned features of rye bread and bread enriched with malt were rated from “I like it quite”—7 to “I like it very much”—8. Statistically significant differences were noted only when assessing the general external appearance of the bread and crust color. In terms of these features, the bread with 9% WBM was given the lowest number of points (respectively, 5.1 and 4.6), and the bread with 3% RBM was given the highest number of points (respectively, 7.7 and 6.9). It was also noted that breads containing 6% and 9% of WBM and RBM obtained the lowest acceptance of crust color among all the characteristics (from 4.6–9% WBM to 6.0–9% RBM) and general external appearance (from 5.1–9% WBM to 6.5–9% RBM). Their rating corresponds to quality characteristics of 5—“neither like nor dislike” and 6—“moderately like”.

A study by Ukeyima et al. [37] also conducted a consumer evaluation. The authors showed greater differences between the evaluation of the control bread and that of bread with the addition of white bean seeds in terms of crust and crumb color, taste, and general acceptability compared to the differences determined in their own research. Breads with 15 and 20% seeds were rated the best in terms of these quality characteristics. In this case, the use of the additive improved the consumer evaluation results of the bread. However, according to Manonmani et al. [38], as the share of red bean seeds increased, breads became less and less attractive to consumers in terms of taste and structure. The discrepancies in the consumer evaluation results among the study by Manonmani et al. [38], the study by Ukeyima et al. [37], and our own study were probably due to the use of different methods of processing bean seeds before adding them to the flour and differences in the size of the additives used.

## 3. Materials and Methods

### 3.1. Materials

Rye bread was made using type 720 rye flour from mill company Goodmills Polska Sp. z o.o. (Stradunia, Poland) with addition of two types of bean malt: white bean malt (WBM) and red bean malt (RBM). Both types of bean were purchased in local grocery shop. Bean malts were prepared using a malting procedure [25], with the change of the germination time, which was equal to 96 h. WBM and RBM were ground in a laboratory mill (Perten Instruments, Hägersten, Sweden) and used to prepare blends with rye flour. The content of WBM and RBM in flour blends was 3%, 6%, and 9%. Rye flour served as the control (0% WBM or RBM). In the production of bread, evaporated iodized salt produced by Cenos Sp z o.o. (Września, Poland) and fresh baker’s yeast produced by Lesaffre Polska S.A. (Wołczyn, Poland), purchased in a local supermarket, were used.

### 3.2. Chemicals

The compounds Folin–Ciocalteau phenol reagent, 2,2-Diphenyl-1-picrylhydrazyl (DPPH), 2,2′-azinobis-(3-ethylbenzothiazoline-6-sulfonic acid) (ABTS^●+^), 6-hydroxy-2,5,7,8-tetramethylchroman-2-carboxylic acid (Trolox), 2,4,6-tri(2-pyridyl)-s-triazine (TPTZ), boric acid, and sulphuric acid were obtained from Sigma-Aldrich (Steinheim, Germany). Sodium carbonate, hydrochloric acid, bromocresol green, methyl red, Kjedahl tablets (cooper II sulphate + potassium sulfate), and lactic acid (80% solution) were purchased from Chempur (Piekary Śląskie, Poland).

### 3.3. Analysis of Flour, Flour Blends, and Pastes

The falling number of rye flour and its blends with WBM and RBM was determined according to the Hagber–Perten method as specified in PN-EN ISO 3093:2009 [51]. Properties of pastes made from rye flour and its blends with WBM and RBM were determined using an amylograph (Brabender OHG, Duisburg, Germany) according to the AACC method 22–10.01 [52]. Obtained amylograms were used to read out the maximum viscosity.

### 3.4. Laboratory Baking

Rye bread (control sample) and bread with addition of WBM and RBM were baked using a single-stage method according to the recipe presented in Table 7.

The obtained dough was placed in metal baking pans (8.5 × 8.5 cm at the base, 13 × 13 cm at the top edge, 10.5 cm in height) greased with oil. The pans were placed in a fermentation chamber, where the dough was allowed to ferment for 90 min (temperature of 30 °C, relative humidity 85%). Afterward, the dough was manually degassed and left for final fermentation (50–60 min). The bread was baked in two replications in a GT800 electric furnace (IBIS, Szubin, Poland), at a temperature of 260 °C for 35 min, with steaming in the first 3 min of baking.

### 3.5. Quality Evaluation

Cooled breads after 2 h of cooling were weighed to determine bread yield based on the weight of flour used for preparing the loaf. The volume of the breads was measured using a SA-WY bread volumeter (Instytut Sadkiewicza, Bydgoszcz, Poland) filled with millet grain and converted as bread volume per 100 g of flour and specific volume (bread loaf volume divided by bread loaf weight). The color of the bread crumb was measured in the CIE L*a*b* space (Konica Minolta Chroma Meter CR-2000b colorimeter, Ramsey, MN, USA). The organoleptic evaluation was performed by a team of 10 panelists according to the Polish Standard (PN-A-74108:1996) [53]. They were informed about the study’s objectives and provided written consent to conduct the organoleptic analysis. The assessment was made for such attributes as the external appearance of a bread loaf, crust color, crumb color, crumb porosity, crumb consistency, taste, aroma, and overall acceptability using a hedonic scale ranging from 1 to 9 points. The scale was defined as follows: 1—Extremely dislike, 2—Very much dislike, 3—Quite dislike, 4—Moderately dislike, 5—Neither like nor dislike, 6—Moderately like, 7—Quite like, 8—Very much like, and 9—Extremely like.

### 3.6. Chemical Composition of Breads

Moisture content of the bread was determined by the oven-dry method according to PN-EN ISO 712:2012 [54]. The chemical composition of bread was determined by measuring total protein content, with the Kjeldahl method (N × 5.7) using a Foss Tecator Kjeltec 2400 analyzer (Foss, Hilleroed, Denmark); starch content, polarimetrically with the Ewers method (PN-EN-ISO 10520:2002) [55]; lipid content, with the Soxhlet method [56]; ash content, with the AACC method 46.11 A [52]; and total dietary fiber content, according to the AOAC method 985.29 [56] using total dietary fiber assay kits TDF-100A-1KT and TDF-C10 (Sigma-Aldrich, Saint Louis, MO, USA). The results are expressed on a dry matter (d.m.) basis.

Energy value of bread was calculated using the following formula: EV = PT × 4 kcal + SC × 4 kcal + DFC × 4 kcal + LC × 9 kcal, where EV—energy value, PT—protein content, SC—starch content, DFC—dietary fiber content, and LC—lipid content.

### 3.7. Amino Acid Analysis

The amino acid composition of samples was determined by ion-exchange chromatography after 23 h hydrolysis with 6 N HCl at 110 °C. After cooling, filtering, and washing, the hydrolyzed sample was evaporated in a vacuum evaporator at a temperature below 50 °C. The dry residue was dissolved in a buffer of pH 2.2. The prepared sample was analyzed using the ninhydrin method [57,58]. The pH 2.6, 3.0, 4.25, and 7.9 buffers were applied. The ninhydrin solution was buffered at pH 5.5. The hydrolyzed amino acids were determined using an AAA-400 analyzer (INGOS Ltd., Prague, Czech Republic). A photometric detector was used, working at two wavelengths, 440 nm and 570 nm. A column of 350 × 3.7 mm, packed with ion exchanger Ostion LG ANB (INGOS Ltd., Prague, Czech Republic), was utilized. The column temperature was kept at 60–74 °C and the detector at 121 °C. The calculations were carried out relative to an external standard. No analysis of tryptophan was carried out.

### 3.8. Polyphenol Content and Antioxidant Activity

The crumb extracts were prepared according to the Czubaszek et al. (2021) method [59]. The total polyphenol content (TPC) was determined using the Folin–Ciocialteu method [60]. The results were expressed in mg gallic acid equivalent (GAE) per 1 g dry matter (d.m.). The ABTS^•+^ antioxidant activity was determined by ABTS radical cation decolorization assay [61] using the DPPH method according to Gow-Chin Yen and Chen [62] and the FRAP method according to Benzie and Strain [63]. The obtained results were performed in triplicate and expressed in μmol of Trolox per g dry weight (μmol TE/g DW). During total phenolic content and antioxidant activity determination, a Shimadzu UV-2401 PC spectrophotometer (Kyoto, Japan) was used.

### 3.9. Volatile Compounds

To perform chromatographic analysis of volatiles present in the bread, the HS-SPME-GC-MS method described by Gasiński and Kawa-Rygielska [48] was used with modifications consisting of using 1 g of bread instead of 2.5 g malt. Furthermore, neither salt nor water was added, the heatplate temperature was set to 40 °C, adsorption time was 20 min instead of 40 min, and 50 ng of internal standard was applied.

### 3.10. Statistical Analysis

The results were analyzed statistically by Statistica 13.1 software (StatSoft, Kraków, Poland), using one-way analysis of variance (ANOVA). Differences between the mean values were assessed using Duncan’s test at the significance level α = 0.05.

## 4. Conclusions

The present study demonstrated that partial rye flour replacement with white bean malt (WBM) and red bean malt (RBM) did not cause significant changes in the consumer assessment of bread. It can be an effective way to increase the content of protein, essential amino acids, polyphenols, and antioxidant activity of bread without deterioration in the quality. Based on the nutritional, functional, and sensory characteristics, it is recommended to consume bread with 6% and 9% addition of white or red bean malt. By assessing the content of volatile compounds in the tested breads, 34 compounds were identified, and it was found that the additives used increased their amount, which may have a beneficial effect on the aroma and taste of the finished product with WBM and RBM. It is planned to conduct further research on the impact of the addition of malt from the seeds of various legumes as a factor enriching the nutritional value of rye bread, using various dough preparation methods. It will also be important to determine the interactions between the dough ingredients.

## Figures and Tables

**Table 1:** Average values of qualitative characteristics of rye bread and rye bread with white bean malt (WBM) and red bean malt (RBM) addition.

Rye Bread with Malt Addition		Bread Yield [%]	Bread Volume [cm^3^]	Color					
				Bread Crust			Bread Crumb		
Type of Malt	Malt Content [%]			L*	a*	b*	L*	a*	b*
Control sample	0	160.5 ^a^ ± 0.21	401.7 ^b^ ± 6.9	32.99 ^a^ ± 1.79	9.73 ^a^ ± 0.86	9.14 ^a^ ± 2.81	55.51 ^a^ ± 0.31	2.89 ^d^ ± 0.11	18.62 ^c^ ± 0.33
WBM	3	163.4 ^a^ ± 0.14	414.2 ^ab^ ± 11.0	30.47 ^b^ ± 0.62	7.66 ^b^ ± 0.79	5.08 ^bc^ ± 1.53	56.00 ^a^ ± 0.47	2.95 ^d^ ± 0.14	19.33 ^b^ ± 0.46
	6	161.4 ^a^ ± 0.85	419.2 ^ab^ ± 9.2	29.27 ^b^ ± 0.85	6.14 ^c^ ± 1.30	3.23 ^c^ ± 1.42	55.56 ^a^ ± 0.53	2.93 ^d^ ± 0.06	19.40 ^b^ ± 0.52
	9	162.2 ^a^ ± 3.18	424.2 ^ab^ ± 7.4	30.21 ^b^ ± 2.08	6.60 ^bc^ ± 2.30	4.36 ^bc^ ± 2.81	55.68 ^a^ ± 0.17	2.80 ^e^ ± 0.05	19.63 ^a^ ± 0.17
RBM	3	161.8 ^a^ ± 0.64	420.8 ^ab^ ± 4.2	33.25 ^a^ ± 1.65	9.37 ^a^ ± 0.72	8.47 ^a^ ± 1.67	52.52 ^b^ ± 1.18	3.51 ^c^ ± 0.09	17.93 ^d^ ± 1.09
	6	160.2 ^a^ ± 0.42	422.5 ^ab^ ± 4.2	32.12 ^a^ ± 2.31	7.60 ^b^ ± 2.14	6.06 ^b^ ± 3.48	53.02 ^b^ ± 2.81	3.74 ^b^ ± 0.07	17.28 ^e^ ± 0.33
	9	160.3 ^a^ ± 1.48	431.7 ^a^ ± 10.4	32.88 ^a^ ± 1.62	7.90 ^b^ ± 1.57	6.20 ^b^ ± 2.22	51.17 ^c^ ± 0.45	4.10 ^a^ ± 0.10	16.75 ^f^ ± 0.50

^a–f^—values with different letters in columns were significantly different at *p* = 0.05 according to Duncan’s test. The data are expressed as mean (*n* = 3).

**Table 2:** Average values of moisture, nutrient content, and energetic value of rye bread and rye bread with white bean malt (WBM) and red bean malt (RBM) addition.

Rye Bread with Malt Addition		Water [g/100 g]	Ash [g/100 g]	Fat [g/100 g]	Total Protein [g/100 g]	Starch [g/100 g]	Total Dietary Fiber [g/100 g]	Energetic Value [kcal/100 g]
Type of Malt	Malt Content [%]							
Control sample	0	48.15 ^abc^ ± 0.35	0.73 ^ab^ ± 0.03	0.93 ^ab^ ± 0.09	4.12 ^g^ ± 0.03	36.29 ^a^ ± 0.15	10.26 ^c^ ± 0.16	190.53 ^c^
WBM	3	48.45 ^a^ ± 0.07	0.71 ^ab^ ± 0.02	0.97 ^ab^ ± 0.09	4.39 ^f^ ± 0.04	35.65 ^b^ ± 0.00	10.70 ^b^ ± 0.12	190.29 ^c^
	6	48.35 ^ab^ ± 0.07	0.71 ^ab^ ± 0.03	0.89 ^ab^ ± 0.01	4.65 ^d^ ± 0.00	35.28 ^bc^ ± 0.17	10.73 ^b^ ± 0.05	189.19 ^c^
	9	47.60 ^bc^ ± 0.00	0.73 ^ab^ ± 0.00	1.02 ^a^ ± 0.01	4.98 ^a^ ± 0.02	35.36 ^bc^ ± 0.33	11.36 ^a^ ± 0.12	193.26 ^a^
RBM	3	47.45 ^c^ ± 0.78	0.69 ^b^ ± 0.01	0.94 ^ab^ ± 0.04	4.47 ^e^ ± 0.02	36.28 ^a^ ± 0.17	10.50 ^bc^ ± 1.64	192.46 ^b^
	6	47.35 ^c^ ± 0.21	0.75 ^ab^ ± 0.03	0.87 ^b^ ± 0.02	4.75 ^c^ ± 0.02	36.21 ^a^ ± 0.00	10.69 ^b^ ± 0.22	188.70 ^d^
	9	48.75 ^a^ ± 0.35	0.78 ^a^ ± 0.01	0.90 ^ab^ ± 0.00	4.83 ^b^ ± 0.02	35.06 ^c^ ± 0.00	10.71 ^b^ ± 0.15	189.08 ^d^

^a–g^—values with different letters in columns were significantly different at *p* = 0.05 according to Duncan’s test. The data are expressed as mean (*n* = 3).

**Table 3:** Content of amino acids in rye bread and rye bread with white bean malt (WBM) and red bean malt (RBM) addition.

Amino Acid		Control Sample	Rye Bread with Malt Addition					
			WBM			RBM		
		0%	3%	6%	9%	3%	6%	9%
ASP	[mg/g of sample]	5.36 ^c^ ± 0.64	6.01 ^bc^ ± 0.13	7.23 ^ab^ ± 0.04	8.15 ^a^ ± 1.23	8.03 ^a^ ± 0.02	6.35 ^bc^ ± 0.14	6.42 ^bc^ ± 0.05
THR *		2.48 ^c^ ± 0.02	2.74 ^bc^ ± 0.05	3.17 ^ab^ ± 0.01	3.58 ^a^ ± 0.54	2.45 ^c^ ± 0.00	3.00 ^b^ ± 0.08	3.12 ^ab^ ± 0.06
SER		3.16 ^c^ ± 0.01	3.47 ^bc^ ± 0.07	3.98 ^b^ ± 0.01	4.67 ^a^ ± 0.69	3.14 ^c^ ± 0.01	3.84 ^b^ ± 0.06	3.97 ^b^ ± 0.03
GLU		20.99 ^d^ ± 0.76	21.62 ^d^ ± 0.52	21.72 ^d^ ± 0.26	25.13 ^b^ ± 0.41	21.90 ^d^ ± 0.18	23.53 ^c^ ± 0.14	26.26 ^a^ ± 0.19
PRO		3.80 ^ab^ ± 0.01	2.60 ^c^ ± 0.18	2.23 ^c^ ± 0.08	4.62 ^a^ ± 0.39	4.68 ^a^ ± 0.09	2.97 ^bc^ ± 0.38	4.60 ^a^ ± 0.81
GLY		2.96 ^c^ ± 0.05	3.14 ^bc^ ± 0.08	3.53 ^bc^ ± 0.02	4.30 ^a^ ± 0.68	2.95 ^c^ ± 0.00	3.52 ^bc^ ± 0.04	3.72 ^ab^ ± 0.05
ALA		2.88 ^c^ ± 0.04	3.17 ^bc^ ± 0.06	3.58 ^b^ ± 0.00	4.24 ^a^ ± 0.67	2.91 ^c^ ± 0.02	3.48 ^bc^ ± 0.07	3.61 ^b^ ± 0.00
CYS *		1.03 ^a^ ± 0.03	0.74 ^c^ ± 0.02	0.79 ^c^ ± 0.08	0.97 ^ab^ ± 0.07	0.29 ^d^ ± 0.03	0.75 ^c^ ± 0.05	0.85 ^bc^ ± 0.06
VAL *		3.10 ^c^ ± 0.00	3.50 ^bc^ ± 0.08	4.13 ^b^ ± 0.00	4.87 ^a^ ± 0.76	3.01 ^c^ ± 0.01	3.94 ^b^ ± 0.08	4.12 ^b^ ± 0.06
MET *		1.03 ^cd^ ± 0.03	1.13 ^abc^ ± 0.03	1.15 ^abc^ ± 0.00	1.33 ^a^ ± 0.19	0.96 ^d^ ± 0.00	1.10 ^cd^ ± 0.03	1.31 ^ab^ ± 0.01
ILE *		2.23 ^c^ ± 0.04	2.71 ^bc^ ± 0.05	3.23 ^b^ ± 0.00	3.87 ^a^ ± 0.60	2.44 ^c^ ± 0.00	3.03 ^b^ ± 0.07	3.13 ^b^ ± 0.02
LEU *		4.51 ^d^ ± 0.06	5.21 ^bcd^ ± 0.07	5.96 ^b^ ± 0.02	7.32 ^a^ ± 1.17	4.67 ^cd^ ± 0.01	5.72 ^bc^ ± 0.09	5.98 ^b^ ± 0.05
TYR *		1.49 ^abc^ ± 0.00	1.42 ^bc^ ± 0.03	1.72 ^a^ ± 0.00	1.69 ^a^ ± 0.27	1.09 ^d^ ± 0.00	1.36 ^c^ ± 0.03	1.64 ^ab^ ± 0.02
PHE *		3.39 ^c^ ± 0.00	3.76 ^bc^ ± 0.10	4.38 ^b^ ± 0.00	5.26 ^a^ ± 0.86	3.40 ^c^ ± 0.01	4.31 ^b^ ± 0.10	4.39 ^b^ ± 0.04
HIS		1.52 ^c^ ± 0.02	1.81 ^bc^ ± 0.01	2.04 ^ab^ ± 0.03	2.32 ^a^ ± 0.44	1.69 ^bc^ ± 0.03	1.84 ^bc^ ± 0.03	2.02 ^ab^ ± 0.01
LYS *		2.77 ^d^ ± 0.00	3.20 ^cd^ ± 0.10	3.96 ^b^ ± 0.03	4.76 ^a^ ± 0.72	2.90 ^d^ ± 0.03	3.64 ^bc^ ± 0.16	3.71 ^bc^ ± 0.05
ARG		3.37 ^bc^ ± 0.14	3.59 ^bc^ ± 0.10	4.13 ^ab^ ± 0.01	4.66 ^a^ ± 0.75	3.20 ^c^ ± 0.22	3.63 ^bc^ ± 0.08	4.11 ^ab^ ± 0.04
Σaa		66.09 ^e^ ± 1.53	69.83 ^d^ ± 0.29	76.93 ^c^ ± 0.25	84.77 ^a^ ± 0.26	69.71 ^d^ ± 0.16	76.01 ^c^ ± 0.59	82.95 ^b^ ± 0.15
TP	[g/100 g]	7.96 ^f^ ± 0.22	8.52 ^e^ ± 0.85	8.99 ^d^ ± 1.12	9.51 ^a^ ± 1.14	8.50 ^f^ ± 0.35	9.02 ^c^ ± 0.89	9.43 ^b^ ± 0.74

^a–f^—values with different letters in rows were significantly different at *p* = 0.05 according to Duncan’s test. The data are expressed as mean (*n* = 3); ASP—aspartic acid, THR—threonine, SER—serine, GLU—glutamic acid, PRO—proline, GLY—glycine (Gly), ALA—alanine, CYS—cysteine, VAL—valine, MET—methionine, ILE—isoleucine, LEU—leucine, TYR—tyrosine, PHE—phenyloalanine, HIS—histidine, LYS—lysine, ARG—arginine; Σaa—sum of amino acids; TP—total protein content; * means content of essential amino acids.

**Table 4:** Average values of total phenolic content and antioxidant activity of rye bread and rye bread with white bean malt (WBM) and red bean malt (RBM) addition.

Rye Bread with Malt Addition		Total Phenolic Content [mg GA/100 g]	DPPH [µmol TE/100 g]	FRAP [µmol TE/100 g]	ABTS [µmol TE/100 g]
Type of Malt	Malt Content [%]				
Control sample	0	26.87 ^c^ ± 1.40	0.59 ^c^ ± 0.01	1.76 ^d^ ± 0.06	1.04 ^e^ ± 0.08
WBM	3	30.00 ^b^ ± 1.75	0.62 ^bc^ ± 0.03	1.80 ^d^ ± 0.02	1.13 ^de^ ± 0.02
	6	35.25 ^a^ ± 0.42	0.62 ^bc^ ± 0.03	2.04 ^b^ ± 0.04	1.30 ^bc^ ± 0.05
	9	35.92 ^a^ ± 0.32	0.68 ^b^ ± 0.04	1.92 ^c^ ± 0.05	1.40 ^b^ ± 0.08
RBM	3	29.90 ^b^ ± 1.02	0.75 ^a^ ± 0.06	1.92 ^c^ ± 0.06	1.22 ^cd^ ± 0.09
	6	33.91 ^a^ ± 1.53	0.78 ^a^ ± 0.04	2.26 ^a^ ± 0.04	1.40 ^b^ ± 0.12
	9	35.60 ^a^ ± 1.84	0.81 ^a^ ± 0.03	2.28 ^a^ ± 0.04	1.57 ^a^ ± 0.07

^a–e^—values with different letters in columns were significantly different at *p* = 0.05 according to Duncan’s test. The data are expressed as mean (*n* = 3).

**Table 5:** Volatile compounds of rye bread and rye bread with white bean malt (WBM) and red bean malt (RBM) addition.

Compound		Control Sample	Rye Bread with Malt Addition					
			White Bean Malt			Red Bean Malt		
		0%	3%	6%	9%	3%	6%	9%
1-Hexanol	µg/L	139.20 ± 9.28	148.79 ± 1.76	248.78 ± 33.63	298.75 ± 62.08	388.20 ± 18.17	247.69 ± 65.17	174.57 ± 4.10
Heptanal		4.71 ± 0.31	6.86 ± 2.18	23.15 ± 4.11	29.38 ± 16.19	32.70 ± 0.50	23.25 ± 5.21	18.92 ± 2.26
2-Heptenal, (E)-		5.48 ± 0.37	4.79 ± 1.24	6.03 ± 0.23	6.75 ± 2.43	7.20 ± 0.57	4.81 ± 1.13	3.21 ± 0.46
Benzaldehyde		8.80 ± 0.59	10.26 ± 1.81	12.18 ± 2.03	15.87 ± 9.35	20.99 ± 2.57	14.96 ± 2.91	8.72 ± 1.67
1-Heptanol		6.91 ± 0.46	6.61 ± 0.21	7.41 ± 1.22	8.44 ± 3.46	11.07 ± 0.27	8.15 ± 1.49	5.43 ± 0.21
1-Octen-3-ol		7.82 ± 0.52	13.04 ± 0.29	19.75 ± 0.45	32.08 ± 11.23	15.54 ± 0.83	10.23 ± 3.03	8.37 ± 0.63
2,3-Octanedione		14.90 ± 0.99	19.66 ± 0.52	10.83 ± 1.35	14.86 ± 6.64	29.55 ± 8.66	22.89 ± 6.09	20.06 ± 1.18
Furan, 2-pentyl-		97.98 ± 6.53	106.98 ± 2.16	107.32 ± 11.68	116.96 ± 50.71	162.04 ± 8.86	135.41 ± 40.73	87.05 ± 12.62
Hexanoic acid, ethyl ester		8.34 ± 0.56	9.88 ± 0.67	9.89 ± 0.02	10.56 ± 5.02	18.18 ± 0.18	12.13 ± 4.06	10.87 ± 0.56
Octanal		9.53 ± 0.64	14.66 ± 0.82	9.05 ± 0.65	12.52 ± 5.97	17.60 ± 0.78	10.74 ± 3.05	6.89 ± 1.10
Acetic acid, hexyl ester		4.68 ± 0.31	6.12 ± 0.08	6.24 ± 1.06	7.61 ± 4.93	10.95 ± 2.02	5.78 ± 2.24	3.93 ± 0.18
1-Hexanol, 2-ethyl-		3.18 ± 0.21	5.90 ± 3.73	29.80 ± 11.68	31.33 ± 13.42	17.43 ± 2.71	16.99 ± 7.97	8.22 ± 1.45
1,3-Hexadiene, 3-ethyl-2-methyl-		8.80 ± 0.59	11.96 ± 1.51	16.47 ± 2.43	16.44 ± 8.26	22.37 ± 1.95	15.68 ± 4.62	9.32 ± 1.36
Benzeneacetic acid, hexyl ester		5.31 ± 0.35	4.80 ± 0.22	5.53 ± 0.41	7.90 ± 3.22	8.29 ± 0.25	8.27 ± 2.44	3.93 ± 0.40
2-Octenal, (E)-		8.34 ± 0.56	9.22 ± 0.72	10.11 ± 0.10	11.16 ± 5.18	14.75 ± 2.66	9.28 ± 3.26	4.45 ± 0.41
1-Octanol		4.89 ± 0.33	4.55 ± 0.80	3.22 ± 0.85	4.21 ± 3.46	6.01 ± 1.11	6.89 ± 5.38	3.20 ± 0.71
Nonanal		31.10 ± 2.07	33.63 ± 0.99	34.98 ± 3.54	47.31 ± 11.64	60.02 ± 11.36	28.46 ± 34.93	25.04 ± 2.75
Phenylethyl Alcohol		5.65 ± 0.38	5.93 ± 0.19	7.47 ± 0.35	13.42 ± 2.40	12.21 ± 0.19	20.74 ± 11.29	4.68 ± 0.39
trans-2-Nonenal		4.92 ± 0.33	3.91 ± 0.10	5.42 ± 0.98	6.82 ± 1.77	6.74 ± 0.51	6.16 ± 0.02	2.63 ± 0.82
Benzenemethanol,α,α,4-trimethyl-		1.95 ± 0.13	1.54 ± 0.11	3.79 ± 4.31	0.00 ± 0.00	0.47 ± 0.67	2.53 ± 2.09	0.88 ± 0.14
Octanoic acid, ethyl ester		6.53 ± 0.43	7.25 ± 0.71	8.41 ± 1.69	10.31 ± 4.66	12.36 ± 0.17	9.32 ± 2.70	7.34 ± 1.46
Decanal		10.37 ± 0.69	9.50 ± 0.40	10.90 ± 1.98	23.82 ± 15.36	45.77 ± 5.41	26.18 ± 6.03	14.33 ± 0.89
2-Decenal, (E)-		2.65 ± 0.18	4.12 ± 0.09	2.52 ± 0.27	2.89 ± 1.05	2.03 ± 2.87	2.65 ± 0.96	1.19 ± 0.24
Nonane, 5-(2-methylpropyl)-		4.33 ± 0.29	3.73 ± 0.10	4.49 ± 1.47	3.61 ± 2.16	6.18 ± 0.14	5.82 ± 1.33	4.72 ± 0.86
2-Undecanal		0.00 ± 0.00	0.00 ± 0.00	0.00 ± 0.00	0.00 ± 0.00	2.89 ± 0.35	2.29 ± 0.97	1.50 ± 0.04
2,4-Decadienal, (E,E)-		10.12 ± 0.67	10.03 ± 1.33	10.26 ± 0.72	14.46 ± 5.92	12.81 ± 3.19	10.24 ± 1.03	4.84 ± 0.07
Dodecane, 4,6-dimethyl-		4.22 ± 0.28	3.11 ± 0.33	4.23 ± 1.01	3.18 ± 1.77	5.67 ± 0.42	5.28 ± 0.84	3.98 ± 1.06
Decanoic acid, ethyl ester		2.58 ± 0.17	2.55 ± 0.02	2.07 ± 0.48	3.46 ± 1.95	2.99 ± 0.50	2.48 ± 0.71	1.36 ± 0.54
Tetradecane		5.90 ± 0.39	4.36 ± 0.30	4.59 ± 0.20	5.39 ± 1.64	5.91 ± 0.96	5.39 ± 0.81	4.61 ± 0.56
Dodecanal		1.99 ± 0.13	2.55 ± 0.02	2.32 ± 0.39	4.46 ± 1.39	4.58 ± 0.41	3.70 ± 0.60	3.25 ± 0.79
5,9-Undecadien-2-one, 6,10-dimethyl-, (Z)-		3.63 ± 0.24	5.70 ± 1.55	1.60 ± 0.00	2.72 ± 0.44	7.59 ± 1.70	5.93 ± 1.31	3.29 ± 0.12
Pentadecane		5.72 ± 0.38	3.89 ± 0.79	2.84 ± 1.13	2.89 ± 0.61	2.73 ± 0.28	2.90 ± 0.61	2.28 ± 0.24
Tridecanal		0.00 ± 0.00	0.00 ± 0.00	1.50 ± 0.03	2.20 ± 0.83	2.20 ± 0.90	2.58 ± 0.33	2.37 ± 0.66
Tetradecanal		0.00 ± 0.00	0.00 ± 0.00	0.00 ± 0.00	0.00 ± 0.00	1.94 ± 1.27	2.50 ± 0.56	1.20 ± 1.15
total		440.54 ^c^ ± 29.36	485.86 ^bc^ ± 11.20	633.17 ^bc^ ± 80.62	771.77 ^ab^ ± 263.46	977.96 ^a^ ± 19.18	698.28 ^abc^ ± 190.50	466.63 ^bc^ ± 38.81

^a–c^—values with different letters in rows were significantly different at *p* = 0.05 according to Duncan’s test. The data are expressed as mean (*n* = 3).

**Table 6:** Consumer assessment of rye bread and rye bread with white bean malt (WBM) and red bean malt (RBM) addition in 9-point hedonic scale.

Rye Bread with Malt Addition		General External Appearance	Color		Crumb Texture (Porosity)	Crumb Consistency (Hardness)	Smell	Taste	Overall Desirability
Type of Malt	Malt Content [%]		Crust	Crumb					
Control sample	0	7.4 ^ab^	6.9 ^a^	7.9 ^a^	6.8 ^a^	7.3 ^a^	6.8 ^a^	7.2 ^a^	7.2 ^a^
WBM	3	6.7 ^abc^	6.3 ^a^	7.9 ^a^	7.2 ^a^	7.2 ^a^	7.1 ^a^	7.5 ^a^	7.4 ^a^
	6	5.7 ^bc^	5.1 ^a^	7.8 ^a^	7.4 ^a^	7.3 ^a^	7.1 ^a^	7.3 ^a^	7.3 ^a^
	9	5.1 ^c^	4.6 ^a^	7.8 ^a^	7.2 ^a^	7.1 ^a^	6.6 ^a^	6.7 ^a^	6.7 ^a^
RBM	3	7.7 ^a^	6.9 ^a^	7.3 ^a^	7.1 ^a^	6.9 ^a^	6.9 ^a^	6.8 ^a^	7.2 ^a^
	6	5.9 ^abc^	5.3 ^a^	7.0 ^a^	7.3 ^a^	7.4 ^a^	7.2 ^a^	7.4 ^a^	6.9 ^a^
	9	6.5 ^abc^	6.0 ^a^	6.8 ^a^	7.0 ^a^	7.3 ^a^	6.8 ^a^	6.9 ^a^	6.9 ^a^

^a–c^—values with different letters in columns were significantly different at *p* = 0.05 according to Duncan’s test. The data are expressed as mean (*n* = 3).

**Table 7:** Used dough recipe.

Ingredients		Rye Bread (Control Sample)	Rye Bred with Addition of WBM or RBM:		
			3%	6%	9%
rye flour	[g]	300	291	282	273
WBM or RBM	[g]	-	9	18	27
salt	[g]	6.0	6.0	6.0	6.0
compressed yeast	[g]	3.4	3.4	3.4	3.4
lactic acid	[cm^3^]	4.7	4.7	4.7	4.7
water *	[cm^3^]	247	249	252	254

*—The dough was prepared in a farinograph mixer by adding water with a temperature of 30 °C, in an amount ensuring a dough consistency of 250 FU.

## Data Availability

The original contributions presented in this study are included in the article/Supplementary Materials. Further inquiries can be directed to the corresponding author.

## Supplementary Materials

The following supporting information can be downloaded at: https://www.mdpi.com/article/10.3390/molecules30051006/s1, Figure S1: Falling number (a) and maximum viscosity of paste (b) of rye flour and rye flour with white bean malt (WBM) and red bean malt (RBM) addition.
