# Supplementary material for: Nutritional Quality of Rye Bread with the Addition of Selected Malts from Beans
Source: Molecules. 2025 Feb 21;30(5):1006. doi: 10.3390/molecules30051006 (PMC11901539; doi:10.3390/molecules30051006)
Supplement: Supplementary file 1 [file molecules-30-01006-s001.zip › Supplementary material.pdf]

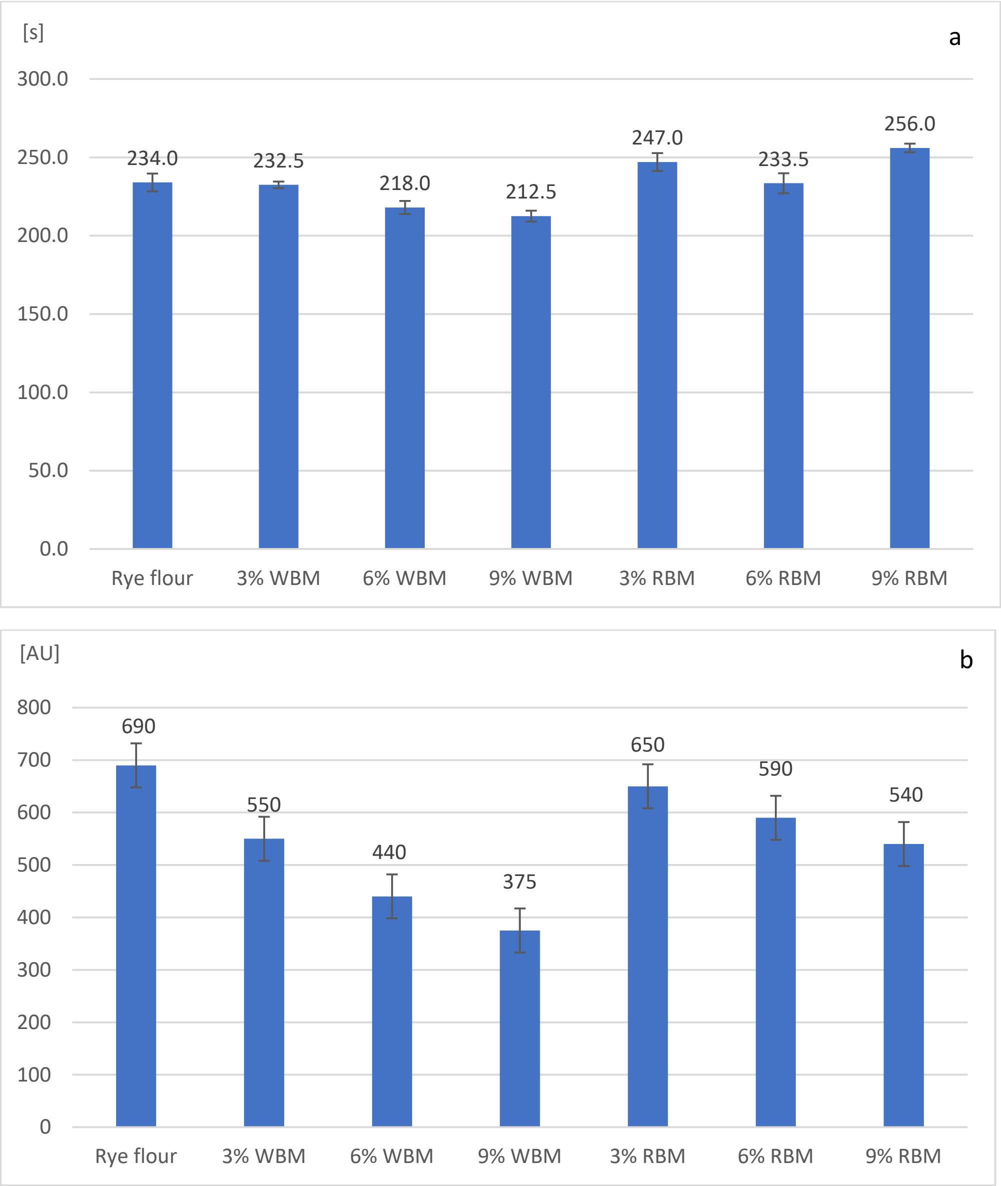

Figure S1. Falling number [s] (S1a) and maximum viscosity of paste (AU)(S1b) of rye flour and rye flour with white bean malt (WBM) and red bean malt (RBM) addition. The data are expressed as mean (n = 3).
